# Attitudes towards Lung Cancer Screening in an Australian High-Risk Population

**DOI:** 10.1155/2013/789057

**Published:** 2013-07-15

**Authors:** Alexandra E. Flynn, Matthew J. Peters, Lucy C. Morgan

**Affiliations:** Department of Thoracic Medicine, Concord Hospital, Hospital Road, Concord, NSW 2139, Australia

## Abstract

*Objectives*. To determine whether persons at high risk of lung cancer would participate in lung cancer screening test if available in Australia and to elicit general attitudes towards cancer screening and factors that might affect participation in a screening program. *Methods*. We developed a 20-item written questionnaire, based on two published telephone interview scripts, addressing attitudes towards cancer screening, perceived risk of lung cancer, and willingness to be screened for lung cancer and to undertake surgery if lung cancer were detected. The questionnaire was given to 102 current and former smokers attending the respiratory clinic and pulmonary rehabilitation programmes. *Results*. We gained 90 eligible responses (M:F, 69:21). Mean [SD] age was 63 [11] and smoking history was 32 [21] pack years. 95% of subjects would participate in a lung cancer screening test, and 91% of these would consider surgery if lung cancer was detected. 44% of subjects considered that they were at risk of lung cancer. This was lower in ex-smokers than in current smokers. *Conclusions*. There is high willingness for lung cancer screening and surgical treatment. There is underrecognition of risk among ex-smokers. This misperception could be a barrier to a successful screening or case-finding programme in Australia.

## 1. Introduction

There is considerable interest in the potential of lung cancer screening using low-dose CT scans to detect nodules that might be lung cancer early, at a treatable time point. Annual spiral CT screening detects lung cancers that are curable [1]. Whilst smaller randomised studies have not shown reduction in mortality, the largest performed to date, the US National Lung Cancer Screening Trial (NLST), showed reduction in both lung cancer specific and total mortalities [2] and has led to recommendations in favour of screening in the USA [3]. If these data are confirmed with other studies presently in progress, the case for CT screening implementation will further strengthen. 

These programs are perhaps more aptly called case detection, rather than screening, as the intervention is targeted at individuals at high risk. The key factors that were used to select patients for inclusion in the research screening programs were age and cumulative smoking history with prolonged period since smoking cessation as an exclusion criterion [2, 4]. Inclusion criteria could be further refined in order to limit the number of subjects screened and increase the rate of cancer detection. The effectiveness of screening will be highly dependent on personal risk recognition by those in the target group.

Other factors that have been used to identify a screened population at higher risk include family history of cancer, asbestos exposure, or previous pneumonia [5]. Abnormal lung function and chronic cough are risk factors for lung cancer independent of smoking history [6, 7]. Abnormal spirometry and CT-diagnosed emphysema both increase the likelihood of lung cancer detection in a screening program [8]. Only lack of data for lung function in the large screened populations prevents the inclusion of lung function in deployed algorithms. Large multimodality screening programs tend to recruit healthier volunteers [9], and this may not be desirable for efficient lung cancer screening. An efficient screening program will attract and include those at the highest risk of lung cancer without severe comorbidities that would preclude curative treatment. 

Australia has been successful in dramatically reducing the prevalence of smoking with adult smoking rates now around 16% [10] with a comprehensive approach including mass media campaigns that have highlighted tobacco use as a cause of lung cancer and do increase quit attempts [11]. Lung cancer remains the commonest cause of cancer mortality in both men and women [12], but a recent community survey found that this was not understood, and other cancers including breast cancer, prostate cancer, and skin cancer were thought to be more common causes of cancer death [13]. 

We reasoned that patients with smoking-related lung disease who attend a teaching hospital would have many of the defining characteristics for lung cancer risk. The aim of this study, therefore, was to explore attitudes of that group towards screening for lung cancer. 

## 2. Methods

### 2.1. Development of Questionnaire

We developed a 20-item written questionnaire (see the Appendix) based in large part on two published telephone interview scripts. From a telephone survey in the USA conducted by Silvestri et al. (with permission) [14], we adapted nine questions about age, gender, general health, previous cancer, and cigarette smoking from a telephone interview format into written tick-box items. We also included four yes/no questions from the telephone interview about each subject's perceived risk of lung cancer: whether a doctor had ever told them that they were at high risk for lung cancer, whether they would consider having a CT scan to determine the presence of lung cancer, and whether they would consider surgery for treatment.

From an Australian survey by Livingston et al. (with permission) [15], we used three questions that relate to perceptions about the benefits of early detection on life expectancy and treatment options [7]. We added one question to determine participation in other cancer screening programs and three new questions to determine whether test accuracy or the cost of a screening test would affect participation.

### 2.2. Subjects

In order to identify subjects at high lung cancer risk, we recruited a convenience sample of patients who were attending the Department of Respiratory Medicine at Concord Hospital or who were participating in the Pulmonary Rehabilitation program. In order to maximise participation, we sought to make the process as simple as possible, and subjects were given the option of self-completion of the questionnaire, home completion and return by post, or completion by telephone.

All subjects provided informed consent for participation, and the study was approved by the Concord Hospital Institutional Ethics Committee.

## 3. Results

### 3.1. Demographics and Background

Completed questionnaires were available for analysis from 91/102 subjects who agreed to participate and were provided a questionnaire. Data from one subject were excluded because of a previous lung cancer diagnosis. 69 subjects were male. Average age [SD] was 68 [11] years with an average cumulative smoking history of 43 [14] pack years. 22% were current smokers. Self-rated health status of the group, prevalence of previous cancers, and participation in other screening tests are shown in Table 1. 75% of women had undergone screening mammography, and 47% of men reported screening for prostate cancer.

### 3.2. Attitudes towards the Early Detection of Cancer

A substantial majority of subjects believed that the early detection of cancer saves lives always or most of the time and that early detection of cancer enabled more effective treatment for lung cancer. Almost all subjects indicated that the chance of surviving would be higher if lung cancer were detected at an early stage (Table 2).

### 3.3. Recalled Information and Perceived Risk of Developing Lung Cancer

A minority of subjects in the whole group thought that they were at risk for lung cancer and even fewer recalled a doctor or another health professional telling them that they were at high risk of lung cancer (Table 3). Overall, former smokers were less likely than current smokers to consider themselves to be at risk for lung cancer (*P* < 0.002 for chi-squared test), and the perceived risk was lower for exsmokers at all smoking intensities. Amongst those subjects with a greater than 20 pack-year history of smoking, who might be a screening target group, half thought that they were at high risk of lung cancer and a third remembered a doctor telling them that they were at high risk. 

### 3.4. Willingness to Undergo Screening and Intervention

There was a high level of willingness to participate in screening (Table 4). All of the four subjects who would not undergo lung cancer screening were current smokers. Test accuracy at two levels had a relatively small effect on intent to participate, and a cost of A$250 (UK *£*160, €200) was not a barrier for the great majority of those who expressed a willingness to undergo screening. Almost all subjects would have lung cancer surgery if offered for a screen-detected tumour.

## 4. Discussion

If the data from the NLST are confirmed, CT-based screening or case detection could have a significant impact on mortality in those at increased risk of lung cancer. There are many preconditions beyond agreement that an effect exists and that modelling for cost effectiveness is favourable. One such precondition is that the target group, once defined, will recognise their risk situation and attend for screening. These data raise important questions about perception of risk in the target group whilst confirming that beliefs in the value of screening and expressed willingness to participate are generally high. 

As interest develops in refining the ideal population for lung cancer case detection, it is important to focus first on the attitudes of those at the greatest risk of lung cancer and the greatest potential benefit. This study was not designed as a whole population sampling study but was conducted in such a population deliberately enriched for lung cancer risk. The difference in attitudes between this population and a more random population sample is uncertain. However, the quality of these data is enhanced by very high participation rate that was, we believe, a product of the study's simplicity. Requiring additional procedures such as current lung function testing may have yielded more data at the expense of participation. 

The average age of subjects, 68, is less than that for lung cancer currently diagnosed in Australia [12]. The median age in the whole population is over 70. However, given that age can indirectly influence the feasibility of curative treatment and that the majority of subjects were in the NLST target age range, this should not compromise the data. Cumulative smoking history is high and similar to that seen in the large lung cancer screening studies [2]. There seems to be no issue with the belief in the value of screening and willingness to participate in lung cancer screening specifically. Complete participation could not be expected and is not seen in bowel cancer screening programs [16] or for mammography [17].

Compared to a more general population in the US study on which this survey was partly based, these subjects had stronger beliefs in the value of screening, are more willing to consider computed tomography screening for lung cancer, and are more likely to opt for surgery if a cancer is detected. This may reflect a true difference in community health beliefs compared to those of the USA but we cannot exclude that beliefs and attitudes of those in the wider community with lesser contact with teaching hospital are different.

We have not tested the desire for lung cancer screening in those at low risk of lung cancer, but this would be important. Based on the major lung cancer screening studies, low-intensity smokers, those who have quit smoking more than 15 years ago and those younger than 50 or 55, would be excluded, but some with those characteristics might wish to be screened. It is reasonable to assume that the detection rate will be lower, that the proportion of benign to malignant nodules requiring further investigation will be higher, and that the radiation risks will be greater, particularly if screening is extended to younger age groups.

Notwithstanding all the public information available, some current smokers and the majority of exsmokers, in contact with tertiary-level health care, do not identify themselves as being at high risk of lung cancer. This is not for want of information. In Australia, general health warnings on cigarette packs were introduced in 1973, text warnings specific to lung cancer were required since 1987, and graphic lung cancer images were incorporated into cigarette packs since 2006 [18]. This study preceded the implementation of plain packaging and enhanced graphic imagery on cigarette packs. In addition, there have been mass media counter advertising campaigns specifically highlighting lung cancer for 40 years with graphic TV advertisements since 1997.

Two factors may contribute to this low-risk assessment. The first is self-exemption. This is a common phenomenon in long-term smokers who underestimate the effect of smoking on lung cancer risk and/or identify illusory counter factors that they suppose to reduce their individual risk. Self-exemption may be factor in the judgment of the four current smokers who would decline screening. The second is an overestimation of the magnitude or immediacy of the benefit of smoking cessation on lung cancer risk. The effect of this combination is that the precise group we wish to target for lung cancer screening may not identify themselves as being at risk and therefore may not present for voluntary screening. If lung cancer screening is implemented, effective information campaigns will need to include the message that exsmokers remain at risk and require screening for up to 15 years.

This will create an immediate tension. The message that smoking causes lung cancer has been an important part of Australian tobacco control programs. Anecdotal feedback suggests that amongst the new graphic cigarette pack images in Australia, that of Bryan, dying of lung cancer, is one of the most impactful images amongst current smokers [19]. Smokers, who are currently quitting, because of a belief that lung cancer risk is rapidly or completely extinguished by smoking cessation, may have misplaced beliefs but benefit greatly. This benefit may be reduced or countered if lung cancer screening program information campaign, that correctly states that an ex-smoker remains at high risk for some time after cessation, is implemented. 

A majority of this group had not been told or did not recall being told by a doctor that they are at risk of lung cancer. It is possible that knowledge of health harm of smoking had been assumed. Alternatively, positive messaging in relation to smoking cessation may have been used to encourage cessation. More complex messaging from health professionals who do talk about smoking and lung cancer will face the same conundrum as that from mass media.

In summary, current and past smokers, in Australia, are willing to undergo lung cancer screening and subsequent treatment. However, the majority who are at high risk of lung cancer do not identify themselves as being at risk. They represent part of the optimal target group for lung cancer screening. Novel and effective education programs targeting this group will be necessary for optimal delivery of a lung cancer screening program, but these will have to be sympathetic with messaging in current antismoking campaigns.

## Figures and Tables

**Table 1:** Subject characteristics.

Health rating	Excellent	Very good	Good	Fair	Poor	Blank
	2	13	34	32	8	1

Previous cancer	None	Skin alone	Bowel/prostate	Breast/cervical	≥2 cancers	Blank
	71	10	5	0	4	1

Screening tests for cancers	Male: ≥2 tests	Male: one test	Male: none	Female: ≥2 tests	Female: one test	Female: none
	25	17	27	15	2	4

**Table 2:** Attitudes towards the early detection of cancer.

General beliefs	Respondents *n* = 90 (%)
How often would early detection of cancer save lives?	
Never/some of the time	15
Most of the time	39
Always/all of the time	30
Don't know	6
How often does finding cancer early mean that a person can have more effective treatment?	
Never/some of the time	10
Most of the time	35
Always/all of the time	39
Don't know	6
Would a person's chance of surviving be higher if lung cancer were detected at an early stage?	
Not at all higher	1
Somewhat higher	20
Very much higher	45
Depends on person/type of cancer	13
Don't know	11

**Table 3:** 

Subjects who believe that they are at risk for lung cancer		
	Former smokers	Current smokers
<20 pack years	21%	50%
20–40 pack years	30%	83%
40–60 pack years	28%	71%
>60 pack years	55%	100%
Any smoking history	34%	75%
Aged 55–74	29%	72%
Total group (*n* = 90)	43%	

Has a doctor ever told you that you are at high risk for lung cancer?		

Total group (*n* = 90)		29%
>20 pack years (*n* = 76)		33%
Former smokers (*n* = 70)		25%
Current smokers (*n* = 20)		40%

**Table 4:** Willingness to undergo screening.

	Yes	No	Blank
Would you consider having this scan done to determine the presence of lung cancer?	86	4	0
If the scan was 90% accurate	83	2	1
If this scan was 70% accurate	71	12	3
If you had to pay $250 to have the scan	69	16	1
Would you have surgery for treatment? (subjects who would undergo scanning)	78	6	2

## Appendix

### Questionnaire

1. What is your age?
2. … years old
3.  Gender—Male/Female
4. Male
5. Female
6.  In general, compared to other people your age, would you say that your health is
7. Excellent
8. Very Good
9. Good
10. Fair
11. Poor
12.  Has a doctor ever told you that you have had any cancer?
13. Yes
14. No
15.  If you have had cancer, what kind of cancer? (tick all relevant boxes)
16. None
17. Lung cancer
18. Breast cancer
19. Bowel cancer
20. Skin cancer
21. Prostate cancer
22. Cervical cancer
23. Other
24. Have you smoked over 100 cigarettes in your life?
25. Yes
26. No
27. Do you currently smoke cigarettes?
28. Yes
29. No
30. For current smokers—On average how many cigarettes do you smoke per day? For former smokers—On average how many cigarettes did you smoke per day?
31. 0–9 cigarettes
32. 10–19
33. 20–29
34. 30–39
35. 40+ cigarettes
36. For current smokers—For how many years have you smoked this amount? For former smokers—For how many years did you smoke this amount?
37. 0–9 yrs
38. 10–19 yrs
39. 20–29 yrs
40. 30–39 yrs
41. 40–49 yrs
42. 50+ yrs
43. Have you ever had a screening test for any cancers? (tick all relevant boxes)
44. Yes—breast cancer (mammogram)
45. Yes—prostate cancer (blood test)
46. Yes—cervical cancer (pap test)
47. Yes—bowel cancer (stool sample)
48. Yes—skin cancer (skin check)
49. No
50. How often, would you say, that early detection of cancer saves lives?
51. Always
52. Most of the time
53. Some of the time
54. Never
55. Don't know
56.  How often does finding cancer early mean that a person can have more effective treatment?
57. Always
58. Most of the time
59. Some of the time
60. Never
61. Don't know
62.  Would a person's chance of surviving be higher if lung cancer were detected at an early stage?
63. Very much higher
64. Somewhat higher
65. Not at all higher
66. Depends on the person/type of cancer
67. Don't know
68.  Has a doctor or other health professional ever told you that you are at high risk for lung cancer?
69. Yes
70. No

Questions continued on next page

(15)  Do you think that you are at risk for lung cancer?
□ Yes
□ No

A new low dose CAT scan has been developed which can find small cancers in the lung. If this scan finds cancer early, when it is small, the chances of curing the cancer is very high.

(16)  If you were told that you were at risk for lung cancer, would you consider having this scan done to determine the presence of lung cancer?
□ Yes
□ No

The scan is not 100% accurate. Some small fast-growing cancers can be missed and some non-harmful benign nodules might be further investigated when they do not need to be.

(17)  If the scan was 90% accurate would you consider having this scan done to determine the presence of lung cancer?
□ Yes
□ No
(18)  If the scan was 70% accurate would you consider having this scan done to determine the presence of lung cancer?
□ Yes
□ No
(19)  If you had to pay $250 to have the scan, would you consider having this scan done to determine the presence of lung cancer?
□ Yes
□ No
(20)  If this scan showed that you had lung cancer would you consider having surgery for treatment?
□ Yes
□ No
